# The impacts of dog sterilization campaigns in Thailand: understanding changes in dog population dynamics

**DOI:** 10.3389/fvets.2026.1926523

**Published:** 2026-10-27

**Authors:** Brontë Slote, Adrian Lau, Mike Dower, Eleanor M. Rees, Jennika Virhia, Nai Rui Chng, Elly Hiby, Katie Hampson, Elaine A. Ferguson

**Affiliations:** 1 School of Biodiversity, One Health & Veterinary Medicine, College of Veterinary & Life Sciences, University of Glasgow, Glasgow, United Kingdom; 2 Independent Researcher, Hong Kong, China; 3 Jai Dog Rescue, Nong Saeng, Thailand; 4 Department of Social Work & Social Policy, Faculty of Humanities & Social Sciences, University of Strathclyde, Glasgow, United Kingdom; 5 International Companion Animal Management Coalition (ICAM), Cambridge, United Kingdom

**Keywords:** animal welfare, domestic dogs, free-roaming dogs, One Health, population control, sterilization

## Abstract

**Introduction:**

Of the estimated near 10 million dogs in Thailand, most are free roaming, raising concerns about nuisance behaviors, dog welfare, and the transmission of diseases such as rabies. Minimal research examines dog population management in Thailand, particularly beyond the Bangkok area.

**Methods:**

We examine the impact of dog population management (DPM), particularly sterilization campaigns in the form of Catch-Neuter-Vaccinate-Return (CNVR), by the non-governmental organization Jai Dog Rescue in the Thai province of Nakhon Nayok. Using transect data collected by the organization and generalized linear mixed models we investigated whether sterilization effort was associated with changes in (1) free-roaming dogs observed per km; and the presence of (2) sterilized dogs; (3) lactating females and puppies; and (4) welfare-related indicators. We triangulated these results with data from a Knowledge, Attitudes, and Practices (KAP) survey in two districts within Nakhon Nayok.

**Results:**

Jai Dog's DPM effort was associated with changes in the dog population through dog counts and people's perceptions. Dogs observed per km and the presence of puppies decreased significantly (*p* < 0.05) in association with sterilization effort. The KAP survey suggests that, when compared with a control district where Jai Dog had not worked, respondents in the intervention district were more likely to report decreased number of dogs on their street (*p* < 0.001), positive perceptions of dog numbers (*p* = 0.01), and less complaints regarding dogs and nuisance behaviors such as dog bites. Our findings show sustained sterilization coverage up to 3 years following peak sterilization effort. The number of sterilizations conducted each year had a compounding effect on this sterilization coverage, but the number of sterilizations 1 year ago showed the strongest association. Furthermore, KAP survey respondents in the intervention district reported higher numbers of sterilizations among their owned dogs (*p* < 0.001) and greater preference for humane dog management strategies (*p* < 0.001).

**Discussion:**

DPM effort was associated with meaningful reductions in free-roaming dogs in Nakhon Nayok province. Jai Dog's monitoring approach using transect counts of directly observed free-roaming dogs is operationally useful, providing a quantitative longitudinal measure of their programme impacts. These results suggest that transects are feasible and valuable for other organizations conducting dog population management.

## 1 Introduction

Free-roaming dogs present concerns for not only human health and safety but also dog welfare. Human-dog conflict can emerge surrounding vehicle accidents, hygiene problems, and injury to humans, wildlife, and livestock (1, 2). Dogs are the vector for several zoonotic diseases, and the primary reservoir for rabies, a fatal disease that kills around 59,000 people annually across Africa and Asia, and that is costly for communities, the health sector, and the livestock industry (3). Should a rabies outbreak occur, a large free-roaming and unvaccinated dog population presents a substantial risk (4, 5). Free-roaming dogs often suffer compromised welfare, with high rates of illness, malnutrition and starvation, and abuse, all contributing to high mortality (6). An unmanaged free-roaming dog population increases the scale of these implications for human and dog health and wellbeing.

Dog population management (DPM) encompasses interventions, directed toward owned and unowned dogs, with the goal of reducing unwanted animals and instances of risky or nuisance behavior, while improving dog health and welfare, and community perceptions (7, 8). Humane DPM does not involve culling, but includes interventions such as sterilization, vaccination, veterinary care, rehoming, and promotion of dog ownership responsibilities (8) through public education, access to services and legislation, which is crucial to long-term success (9). Multiple DPM interventions should ideally be used concurrently, although this complicates determining which are most impactful (9).

Catch-Neuter-Vaccinate-Return (CNVR) is a widely used DPM method, which involves catching unowned or community-owned dogs, neutering and vaccinating them, before returning them to where they were found. CNVR helps control reproduction and prevent disease while reducing reproductive-related welfare problems and may increase lifespans and mitigate sex hormone-associated nuisance behaviors (8–11). When free-roaming dogs are accepted by the local community, CNVR is an appropriate option (10). For this reason, CNVR has been widely used in Asia to control dog populations, improve welfare, and decrease bites (10, 12, 13). As sterilization rates increase, dog populations often decrease (10, 14). The impact of CNVR depends on factors including the programme duration, the proportion of dogs sterilized, and immigration of intact dogs (14). Impacts can only be fully understood if effective monitoring and evaluation systems are in place (1). CNVR requires enduring resource commitment; if halted populations will rebound through breeding by intact females. Rebound also results from abandonment of owned dogs and immigration of intact dogs, hence the need for additional interventions targeting owned dogs (10, 15–17). Large-scale sterilization campaigns are typically limited by resources or political and socio-economic barriers (9). Though initial costs of surgical sterilization are high, they diminish the need for ongoing, resource-heavy population control measures, such as hormonal birth control or creating and running shelters, so could bring long-term economic benefits (18, 19).

Currently there are an estimated 9.91 million dogs in Thailand, with 1.6 million considered unowned (20). More than half the owned dogs are allowed to free-roam, with most free-roaming all the time (21, 22). Surveys found that Thai perceptions of the most problematic dog behaviors were noise, urine and fecal waste, disturbing garbage, and attacks (21, 23). Rabies in Thailand is spread by infected dogs, and nearly all reported rabid dogs are unvaccinated and most are free-roaming (24), highlighting how CNVR could address both dog-associated nuisance behaviors and public health risks.

Responsibility for free-roaming dog issues in Thailand is fragmented. Though predominantly lying with the Department of Livestock Development (DLD), public health authorities maintain involvement in zoonoses management. The DLD conducts dog population estimates and is responsible for vaccination, both pre-emptively and following suspected and confirmed human or animal rabies cases. Dog owners have individual responsibility for their dogs, as keeping them vaccinated and preventing them from biting is legally required (25). A survey in Northern Thailand, showed that most residents believe that the dog population needs to be managed (21), while in surveys across the country respondents identify sterilization as the best way to control numbers of unowned dogs (20, 23). CNVR has been shown to be impactful in the Thai context decreasing dog density and improving positive perceptions of dogs in communities (10, 23).

Our aim was to understand how DPM activities, by the NGO, Jai Dog Rescue, have impacted the dog population and dog welfare in the Thai province of Nakhon Nayok. Jai Dog has been working in the province since 2021 conducting Catch-Neuter-Vaccinate-Return (CNVR), sterilization of owned dogs, and related activities including community education and outreach, rescue and rehabilitation, and support for community dog carers. This analysis was conducted as part of a project to support DPM organizations with monitoring, evaluating, and improving their programmes. The study objectives arose from a collaborative Evaluability Assessment (EA) process working with Jai Dog Rescue to develop their Theory of Change (26). The EA involved a series of workshops where stakeholders were facilitated to reach a shared understanding of their programme's aims, assumptions about how impact is achieved, and to prioritize outcomes that can be evidenced. These priorities directly mapped onto our objectives to assess changes in: (1) free-roaming dogs observed per km, and numbers of (2) sterilized dogs, (3) lactating females and puppies, and (4) sick dogs, over time based on both observed dog population changes and the perceptions of local communities. We hypothesized that in line with Jai Dog's ongoing work, dogs observed per km and the number of lactating females, puppies, and sick dogs would decrease, while neutered dogs would increase. As a result, we would expect communities in areas where Jai Dog works to experience fewer dogs on their streets and negative issues associated with free-roaming dogs, and to have more of their owned dogs sterilized and greater support for humane DPM methods such as CNVR.

## 2 Materials and methods

We use annual transect surveys, alongside a one-time knowledge, attitude, and practice (KAP) survey, to understand how the impact of DPM is reflected in changes in the dog population and community members' perceptions. Transects along public roads were conducted annually in the subdistricts of Khok Kruat and Tha Chang between October 2021, and September 2025 by Jai Dog staff. While Jai Dog's current work focuses on the province of Nakhon Nayok as a whole (Figure 1a), the transect data analyzed is from those subdistricts where Jai Dog has been working the longest and there are the most repeat transects (Figure 1b).

**Figure 1:**
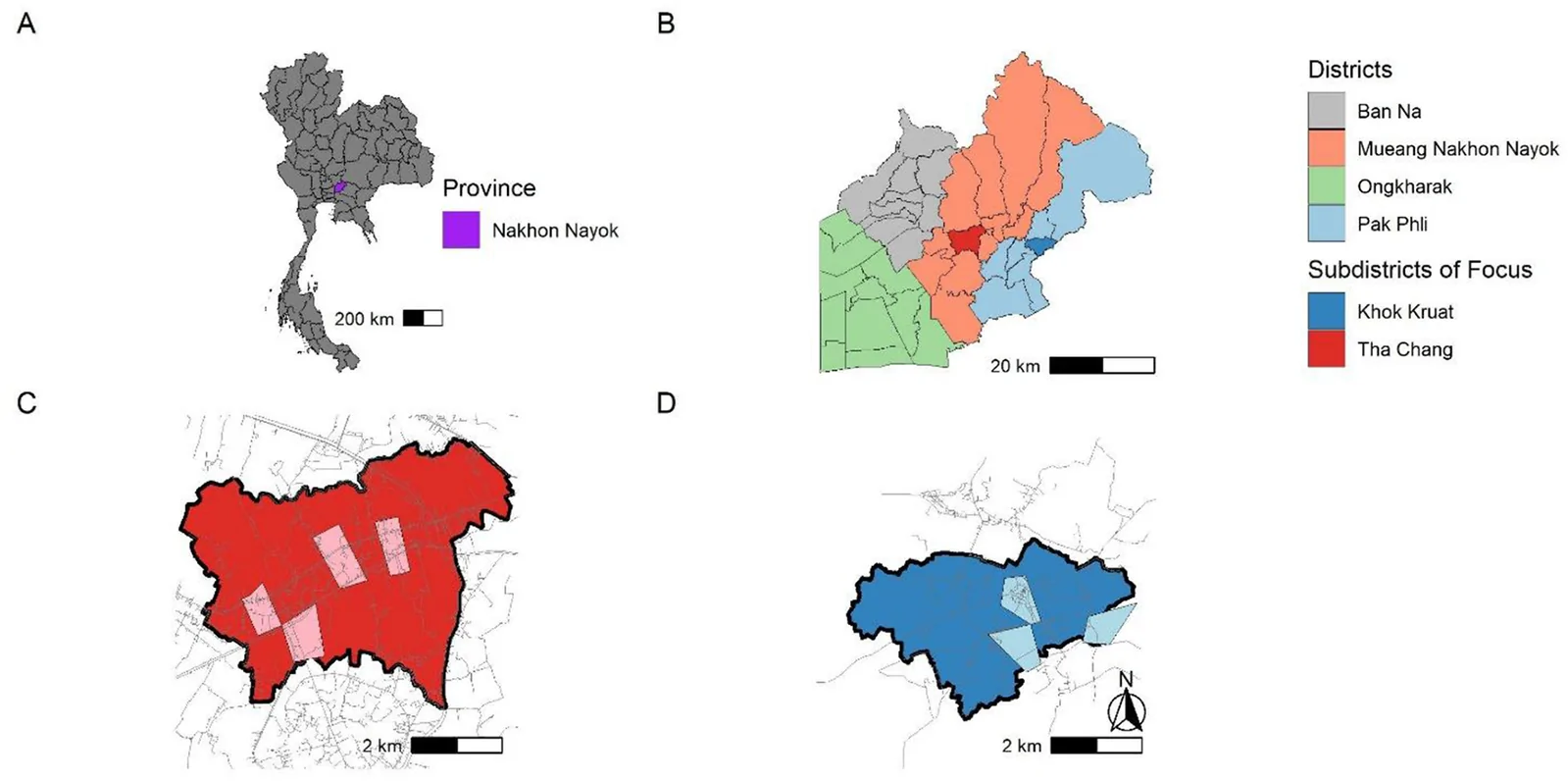
Study Areas in Nakhon Nayok Province, Thailand with subdistricts and survey locations. **(A)** Provinces of Thailand with the province of Nakhon Nayok highlighted. **(B)** Districts of Nakhon Nayok with subdistricts *targeted by the transect surveys* (Khok Kruat and Tha Chang) highlighted. The KAP survey was conducted in the districts of Pak Phli (sterilization intervention present) and Ongkharak (control, no sterilization intervention). **(C)** Tha Chang Subdistrict and transect locations. **(D)** Khok Kruat Subdistrict and transect locations. The polygons used to define the areas covered during transects are outlined in panel C and D. These polygons were drawn and randomly selected by Jai Dog staff using the WVS app when identifying areas for transects that would be walkable within 90 min, these polygons ranged in size between 2.5 and 5 km^2^.

Transect locations were established by dividing each subdistrict in the province into polygons, ranging in size between 2.5 and 5 km^2^, and spanning areas that would be walkable within 90 min. This resulted in a variable number of polygons per subdistrict. Using random selection, three polygons were chosen in Khok Kurat subdistrict (Figure 1c), and six in Tha Chang, although only four polygons from Tha Chang (Figure 1d) were analyzed due to missing data. Within each polygon, transects were defined and surveyed consistently across years. Except for those conducted in 2025 (which were traveled once in a single day), all transects were carried out on two subsequent days. Staff traveled the same transect each day, by foot, bicycle, or vehicle recording dogs seen into an app (https://www.wvsapp.org). Dog age (puppy or adult), sex (male/female/unknown), sterilization status, health and indicators of ownership were recorded based on visual assessment. Sterilization status was determined by ear notches or absence of gonads in male dogs. Dogs were recorded as healthy or unhealthy based on observations by surveyors noting body and skin condition. Puppies were identified as any dog appearing to be 6 months of age or younger, also looking for presence of littermates or mother to indicate age. On the second day, staff also noted any dogs resighted from the previous day. When the identity of a dog was in doubt or ear notching lacking, surveyors communicated with community members and dog catchers to identify dogs, verify sterilization status, and appropriately label resights.

In 2025, a KAP survey was administered in two districts: Pak Phli, the intervention area where Jai Dog had been working since 2022 and in which the subdistrict of Khok Kruat is located, and Ongkharak, the control area where Jai Dog had not been working at the time of the survey (Figure 1b). From each district, two subdistricts were randomly selected. Within subdistricts, households were randomly selected as follows: villages were randomized, a transect was followed through each, and every 2nd or 3rd household was approached regardless of dog ownership, until either the end of the transect was reached or one-quarter of the subdistrict's quota (125 households in each district, around 63 responses per subdistrict) was completed, ensuring at least four villages were sampled per subdistrict. Consent was obtained from participants before commencing the survey, with an adult required to be present if the participant was under 18 years of age. Survey questions established respondents' basic demographic information, then focused on dogs in their community, household dog ownership, veterinary care and neutering, community dog problems and safety, and awareness and attitudes surrounding street dogs. Most questions were asked in the context of whether changes had been perceived within the last 12 months, or comparing to before the first COVID-19 lockdown (2020), to help connect memories with a landmark timepoint. Household occupants who had lived in the study area for less than 12 months were excluded from the survey.

DPM effort was assessed differently in the transect and KAP survey analyses. In analyzing the transect data, dog observations were compared against the number of dogs from Khok Kruat and Tha Chang that were sterilized, with sterilization numbers acting as a measure of the intensity of Jai Dog's DPM work. Dogs were sterilized either at mobile clinics conducted on location in the subdistricts or at Jai Dog's permanent, static clinic located at their nearby headquarters (Figure 2). The mobile sterilization clinics ran from February 2022 to March 2022 in Khok Kruat and from November 2023 to January 2024 in Tha Chang. For this analysis we defined the intervention start dates and peak of intervention as February 11, 2022, for Khok Kurat and November 17, 2023 for Tha Chang. Mobile clinics are placed in strategic, central locations in the subdistrict, only moving to a new subdistrict once dog catchers report a sterilization rate of close to 80% of free-roaming dogs accessible in that subdistrict.

**Figure 2:**
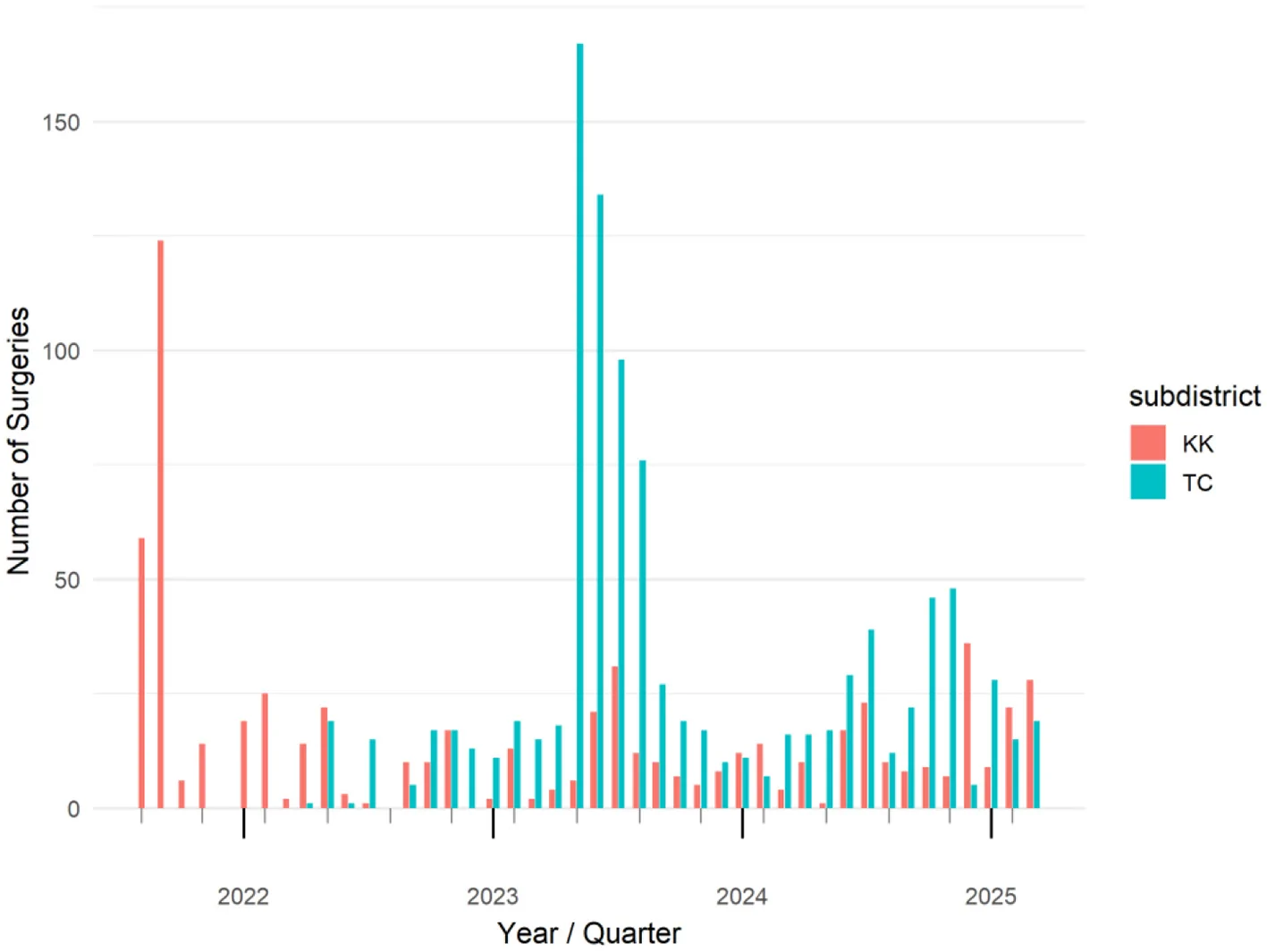
Monthly number of sterilization surgeries conducted from January 2022 to April 2025. The number of sterilization surgeries conducted each month in both Jai Dog's permanent and mobile clinics, with surgeries conducted in the subdistrict of Khok Kruat colored in red and the subdistrict of Tha Chang colored in blue. The peaks in the respective subdistricts' surgery numbers indicate when mobile clinics were present in each area.

After the mobile clinics ended, many community members and Jai dog catchers continued to bring dogs from both subdistricts to be sterilized at the nearby permanent Jai clinic. To account for the dynamics of Jai Dog's sterilization effort (a single high intensity sterilization campaign followed by an ongoing trickle of individual surgeries), our analysis of the transect data explored three approaches for defining sterilization effort: (1) the total number of sterilizations per human capita completed in a subdistrict either across all time (recorded by Jai Dog in the subdistrict), (2) the time that had passed since the peak of the intervention, and (3) the total number of sterilizations per human capita completed 4, 3, 2, and 1 year(s) prior to the transect dates. Measures of total sterilizations and yearly sterilizations were divided by the subdistrict's human population to account for differences in dog population size. We did not have an accurate dog population size estimate; to reflect the impact of the number of sterilizations conducted in each subdistrict we used the human population size recognizing that human density is associated with the distribution of dogs (22). Dividing sterilization numbers by the human population assumed that both subdistricts were generally similar in their overall makeup and would thus have similar human-to-dog ratios. While the transects are divided by polygons, the clinic data is based on the municipal divisions of subdistrict and village. Therefore, generalizations can only be made at a subdistrict level across the data sets. In the KAP survey, DPM effort was indicated by responses being from either within the intervention area where Jai Dog had worked since 2022 or control area where the organization had not been present. The proportion of sterilized owned dogs in each district was determined by survey respondents reporting their owned dogs' sterilization status. It was assumed for the purpose of this analysis that the two districts were relatively similar in their demographic makeup and would serve as accurate comparisons of the impact of Jai Dog's DPM work.

Analysis of the transect data was conducted using R, version 4.4.1 (27). Data were cleaned and organized using the package tidyverse (28) and generalized linear mixed models (GLMMs) were fitted with the lme4 package (29). For each objective we created GLMMs examining the relationship between our response variables and relevant predictors. From the most complex model, we carried out backward selection using likelihood ratio tests, to determine the most parsimonious model. We repeated the model selection three times for each objective, with a different one of the three effort measures being included in the full model each time to avoid collinearity between explanatory variables. AIC was used to compare the fit of the three final models for each objective and indicate which effort indicator most effectively predicted the response variable. In all models, we included a random effect for polygon that was retained throughout model selection unless the variance was estimated to be zero. For objectives 2–4, where the response variable was at the level of the individual dog, we removed all resights to maintain only unique individual sightings in our dataset. Each model was also separately run with all day 2 dog observations removed, to determine if the same effects could be observed had transects not included a second day of data collection.

The response variable in our dogs observed per km models was the total count of dogs sighted on each transect, each day (sample size of 41 transects). A Poisson error structure was used with the explanatory variables of subdistrict, survey day (1 or 2), and mode of transport, as well as a measure of sterilization effort and a nested random effect of polygon and survey id and an offset of log(transect length) to model dogs per km. We found no evidence of overdispersion when testing the fitted model using the DHARMa package (30), justifying the Poisson assumption.

For our second objective, the response variable was the binary measure of whether an individual dog was sterilized or not. Puppies (too young to be sterilized) and dogs of unknown sterilization status were removed from the sample. A binomial error structure was assumed, with explanatory variables of subdistrict, ownership status (owned or unowned), and sex in addition to a measure of sterilization effort.

We addressed our third objective using two models to examine the relationship between sterilization effort and presence of lactating females (binary response variable of whether a female was lactating or not; puppies and males were excluded from this model), and between sterilization effort and the presence of puppies (binary response variable of whether a dog was a puppy or not). Both models used a binomial error structure and the explanatory variables of subdistrict, ownership status, and a measure of sterilization effort. The random effect of polygon was dropped during model selection as it was found to have no variation.

The final model of dog health status employed a binomial error structure with a response variable of whether an individual dog was healthy or sick based on observed welfare indicators. We used explanatory variables of subdistrict, dog sterilization, age (puppy or adult), sex, ownership, and sterilization effort.

Analysis of the KAP survey was conducted using Python. KAP survey analyses were designed to assess associations between district-level DPM effort and outcomes related to our four objectives with an additional focus on dog population characteristics, dog welfare indicators, community safety, and public attitudes toward DPM. All analyses were exploratory and unadjusted, intended to provide descriptive and inferential context for the multivariable modeling. Continuous variables were compared between districts using Welch's two-sample *t*-test to account for unequal variances. Categorical outcomes were analyzed using Pearson's chi-square test of independence. Fisher's Exact Test was applied where expected cell counts were small. Statistical significance was assessed at the 5% level (α = 0.05).

Ethics approval was received from the University of Glasgow Medical, Veterinary, and Life Sciences Ethics Committee (200240421).

## 3 Results

Our transect analysis included 549 dog observations, with a near equal number of dog sightings from each subdistrict (275 in Khok Kruat, 274 in Tha Chang). Of these dogs, 54% showed signs of ownership (presence of a collar, confined by a lead or in a yard) while 46% were categorized as unowned. Of the dogs sterilized at both the mobile and static clinics most were owned, predominantly kept unconfined (mobile clinic = 55%, static clinic = 52%), followed by free-roaming community-owned dogs (mobile clinic = 44%, static clinic = 39%), and strays (mobile clinic = 2%, static clinic = 9%). Only a small number of dogs observed were lactating (*n* = 9, 2%) or puppies (*n* = 18, 3%). Most dogs were healthy, with only 7% (*n* = 39) identified as sick based on visible indicators.

In the KAP survey a total of 330 households were approached, of these 316 complete responses were completed and included in the final sample. The final sample comprised of 164 respondents from the intervention district, Pak Phli: Khok Kruat (*n* = 88, 28%) and Nongseang (*n* = 76, 24%), and 152 respondents from the control district, Ongkharak: Bang Luk Suea (*n* = 75, 24%) and Ongkharak subdistrict (*n* = 77, 24%). The survey had a non-response rate of 4.24%, with 14 of the households approached declining consent. Of those who declined, half did so because there was no adult present, while the remaining declined for unknown reasons. Five of the declined consent were from the control district and 9 from the intervention district. Nearly all respondents were Buddhist (*n* = 312, 98.7%; 98% in the control district, 99.4% in the intervention district), with only a small number being Muslim (*n* = 1, 0.3%) or not disclosing their religion (*n* = 3, 0.9%). There were slightly more female respondents (*n* = 191, 60%), than males (*n* = 39%). Of those surveyed, 55% owned dogs (44.1% in the control district, 46.3% in the intervention district) and only 16% reported caring for stray or community dogs (13.2% in the control district, 19.5% in the intervention district). The KAP survey suggested that outcomes in the DPM intervention district consistently differed from those in the control. These differences were statistically significant and directionally consistent with improved DPM, enhanced public safety, and more favorable community perceptions. While the respondent populations were similar across the districts when examining the descriptors summarized above, suggesting comparable demographic makeups between the two study areas, the KAP analyses were unadjusted and should therefore be considered exploratory and descriptive while suggesting general trends worthy of further analysis.

### 3.1 Objective 1: free-roaming dogs observed per km

From the transect data we found that subdistrict and sterilization effort were significantly associated with the number of dogs observed per km. All measures of sterilization effort correlated with significant declines in dogs observed (Figure 3). AIC comparisons between models incorporating each measure of sterilization effort showed that quantifying effort as time since intervention or total sterilization effort provided near identical fits to the data, while separately accounting for effort in each of the previous 4 years gave a slightly poorer fit (since intervention AIC: 265.07, total effort AIC: 265.92, annual effort AIC: 270.38). Sterilization effort measure(s) and subdistrict remained as the only significant fixed effects following model selection (Table 1). When refitting the models with day 2 transects removed from the data, all sterilization effort measures and subdistrict remained significant, with effect sizes virtually unchanged (Supplementary Materials 2, Tables 1 and 2).

**Figure 3:**
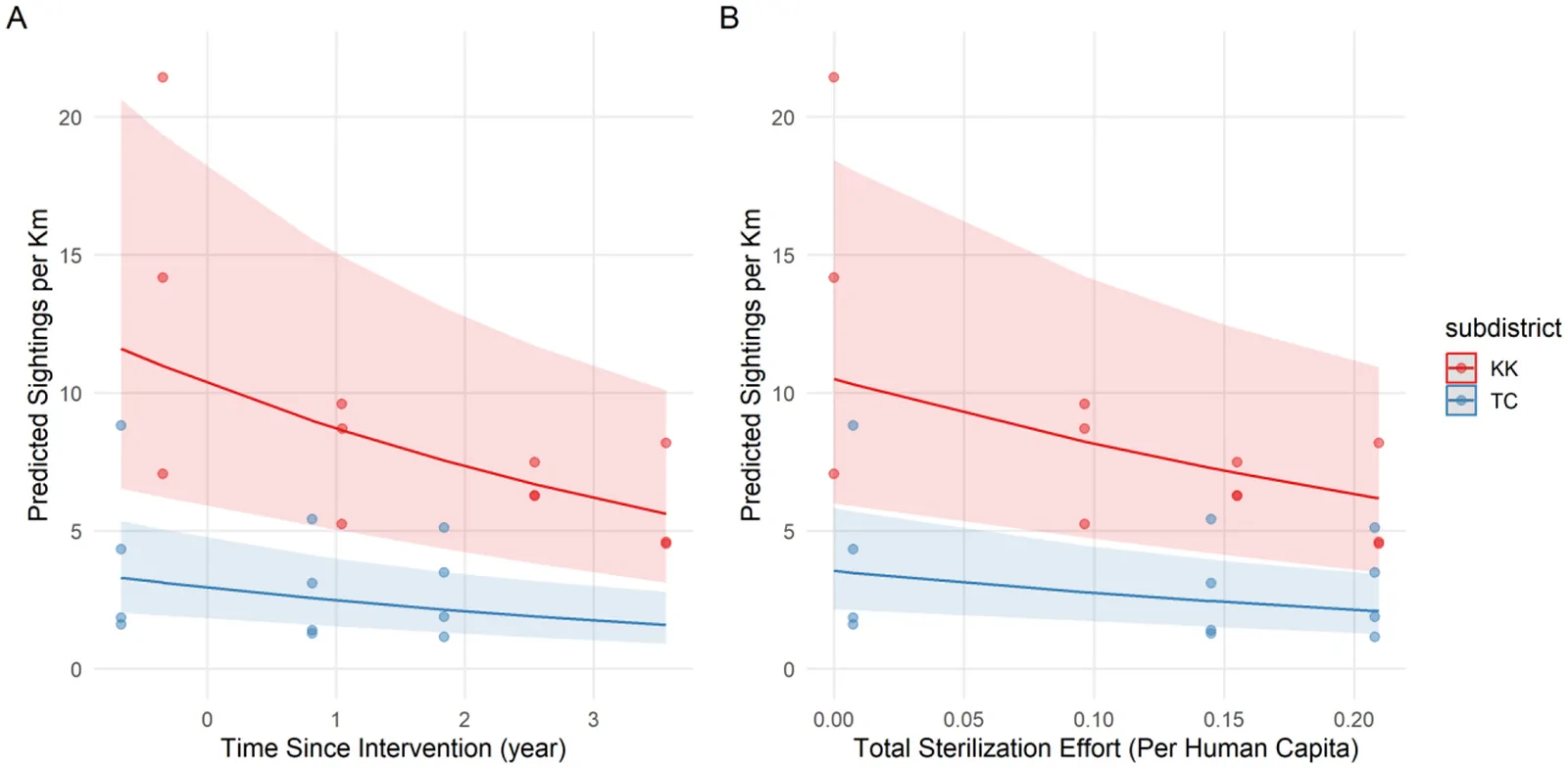
Changes in dog density in relation to time since intervention and total sterilization effort. **(A)** Decline in dogs sighted in association with time since the peak of the intervention, measured in years. **(B)** Decline in dogs sighted in association with the total number of sterilizations conducted up to transect date. Lines demonstrate the model fit with the subdistrict of Khok Kruat colored red, and Tha Chang colored blue. Points mark the average number of dogs observed per km transect, while lines and shaded envelopes indicate estimated means and 95% confidence intervals.

**Table 1:** The best-fitting statistical models examining associations between Jai Dog's sterilization programme and dog density.

		Time since intervention model				Total sterilization effort model			
Parameter type	Variable	Estimate	Standard error	*Z* value	*P*-value	Estimate	Standard error	*Z* value	*P*-value
Fixed effects	Intercept	2.3366	0.2855	8.183	< 0.001	2.3526	0.2871	8.195	< 0.001
	Time since intervention	−0.1712	0.0468	−3.657	0.0003				
	Total sterilization effort					−2.5356	0.7262	−3.491	0.0005
	Subdistrict (Tha Chang)	−1.2553	0.3715	−3.379	0.0007	−1.0850	0.3689	−2.941	0.0033
Random effect	Polygon	0.2100				0.2083			
variance	Survey: Polygon	0.0329				0.0356			

Effort was defined in terms of time since intervention and total sterilization effort. Other models with alternative measures of effort are shown in the Supplementary materials.

Dogs observed per km is estimated to decrease with increasing time since the intervention peak or increasing sterilization effort (Table 1). We also observed major differences between dog numbers in the two subdistricts, with dogs observed per km in Tha Chang lower than Khok Kruat at baseline. At the peak of the intervention, the model using the time since intervention effort measure predicts observing 10.35 dogs/km (95% CI 5.91–18.11) in Khok Kruat, and 2.95 dogs/km (95% CI 1.83–4.74) in Tha Chang. One year after the peak of the intervention the predicted dogs per km in Khok Kruat was 8.72 dogs/km (95% CI 5.04–15.08), and in Tha Chang was 2.48 dogs/km (95% CI 1.54–4.00), continuing to decline over the following years to 5.22 dogs/km (95% CI 2.86–9.52) in Khok Kruat, and 1.49 dogs/km (95% CI 0.84–2.65) Tha Chang after 4 years.

This decline in dogs observed per km was reflected in the KAP survey. Residents in the intervention district reported seeing significantly fewer roaming dogs in their local neighborhood streets (called “sois” in Thailand). In the intervention district, participants reported an average of 2.8 dogs per soi (SD 3.23; median 2, IQR 0–4), vs. an average of 6.1 dogs per soi (SD 10.18; median 4, IQR 0–10) in the control district. Responses from the control contained one extreme estimate of 100 dogs, which was excluded. The unadjusted mean difference between dog counts was −3.31 dogs per soi (95% CI −4.51 to −2.12; exploratory *p* < 0.001). While respondents were not asked to standardize the spatial area considered when estimating dog numbers, the distribution of responses differed notably between districts with respondents from the intervention area reporting a more limited range of dogs counts, mostly between 0 and 4 dogs on their street, whereas there was much more variation in responses in the control with some reports of up to 40 dogs. This indicates that the control area may see larger packs of dogs on their street, in comparison to the intervention area. When asked to consider the number of dogs on their soi today as opposed to pre-COVID, most respondents in the intervention district reported fewer dogs now or the same number, with few reporting more dogs. Whereas in the control district, most respondents said the number of dogs was the same, or had increased, with only a small percentage reporting fewer dogs (Figure 4a).

**Figure 4:**
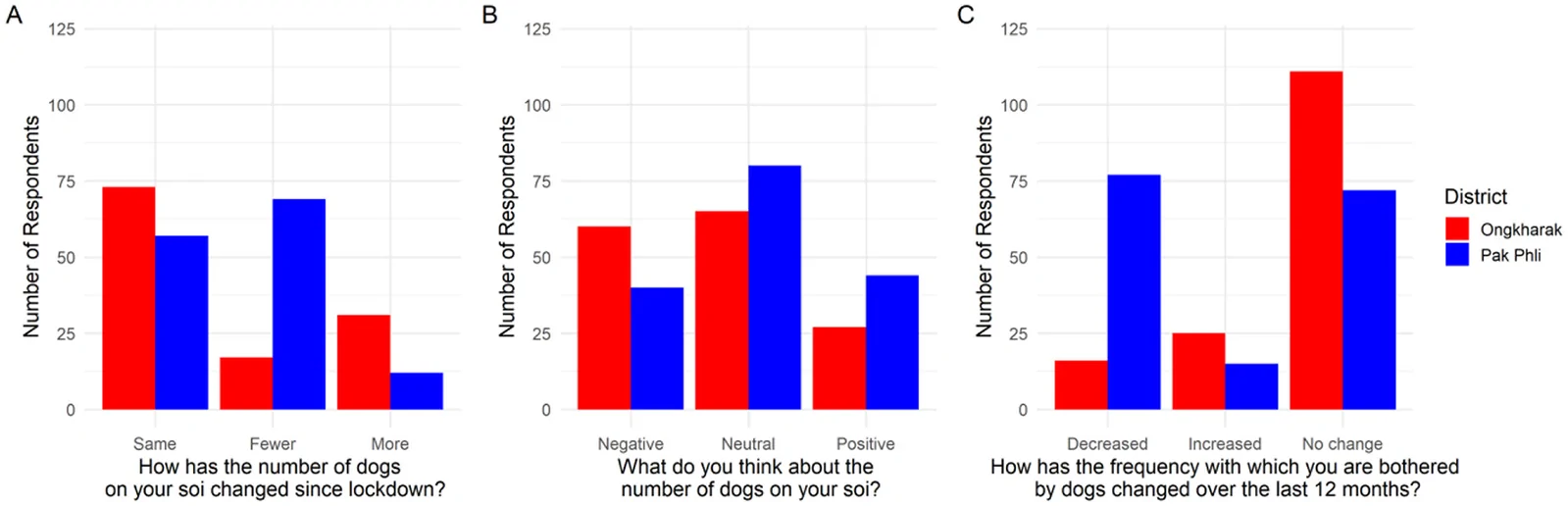
Reported changes in respondents' perceptions of dogs in areas where Jai Dog has worked. **(A)** The number of dogs respondents observed on their street compared to before COVID. **(B)** Respondents' opinions toward the number of dogs on their street, with negative indicating they were unhappy toward the number of dogs on their street, neutral indicating they had no opinion, and positive indicating that they were happy or accepting. **(C)** Change in frequency of respondents being troubled by free-roaming dogs. Red bars correspond to Pak Phli district where the CNVR intervention was carried out, while blue bars correspond to Ongkharak district which was the control area with no intervention.

When asked for their opinion of the number of free-roaming dogs on their soi, most respondents in the intervention district were neutral in their perception of the number of dogs, followed by a positive perception (expressing happiness or acceptance of the number of dogs), with one quarter reporting a negative perception (expressing that they are unhappy or that the number of dogs is unacceptable). In the control district, most respondents were neutral or negative toward the number of dogs on their soi, with less than 20% reporting positive perceptions (Figure 4b). Most participants in the intervention district also reported less trouble with dogs or no change in the last 12 months, whereas most people in the control district reported no change (Figure 4c). The top nuisance behaviors identified across both districts were barking/howling, messing with garbage, and dog fights. In the intervention district, reports of aggressive dog nuisance behaviors were notably fewer and there were fewer dog bites reported compared to the control (1.2 vs. 9.2% of households, exploratory *p* = 0.0013).

### 3.2 Objective 2: sterilized dogs

All measures of sterilization effort were significantly associated with the probability that a dog was sterilized in the transect data. When compared by AIC, sterilization effort by year was the best fit for the data (since intervention AIC: 504.85, total effort AIC: 483.18, sterilization effort by year AIC: 475.96). The number of sterilizations 3 years ago, 2 years ago, and 1 year ago all had a significant positive association with the probability that a dog was sterilized (*p* = < 0.001, Figure 5), while sterilizations effort 4 years ago dropped out. The number of sterilizations conducted 1 year before the survey date had the greatest impact, followed by the number of sterilizations conducted 3 years and 2 years ago (Table 2). With the highest observed sterilization effort 3 years ago, if there were no sterilizations conducted in the subsequent 1 and 2 years, the probability of a female dog being sterilized would be 0.52 (95% CI 0.46–0.58). With the highest observed sterilization effort 1 year ago but no sterilizations in the 2 and 3 years prior, the probability of a female dog being sterilized would be 0.79 (95% CI 0.78–0.81), demonstrating the importance of the more recent sterilizations. Sex was additionally a significant predictor of sterilization status, with female dogs consistently more likely to be sterilized than males. Subdistrict and ownership status were non-significant and dropped from the model during model selection. All measures of sterilization effort, and the predictor of sex, remained significant when the model was tested with day two sightings removed. Sterilization effort 1 year ago and sex remained highly significant across both models (*p* < 0.001), the significance of sterilization effort 3 and 2 years ago declines slightly (*p* < 0.01) with the removal of day 2 data (Supplementary Materials 2, Tables 3 and 4).

**Figure 5:**
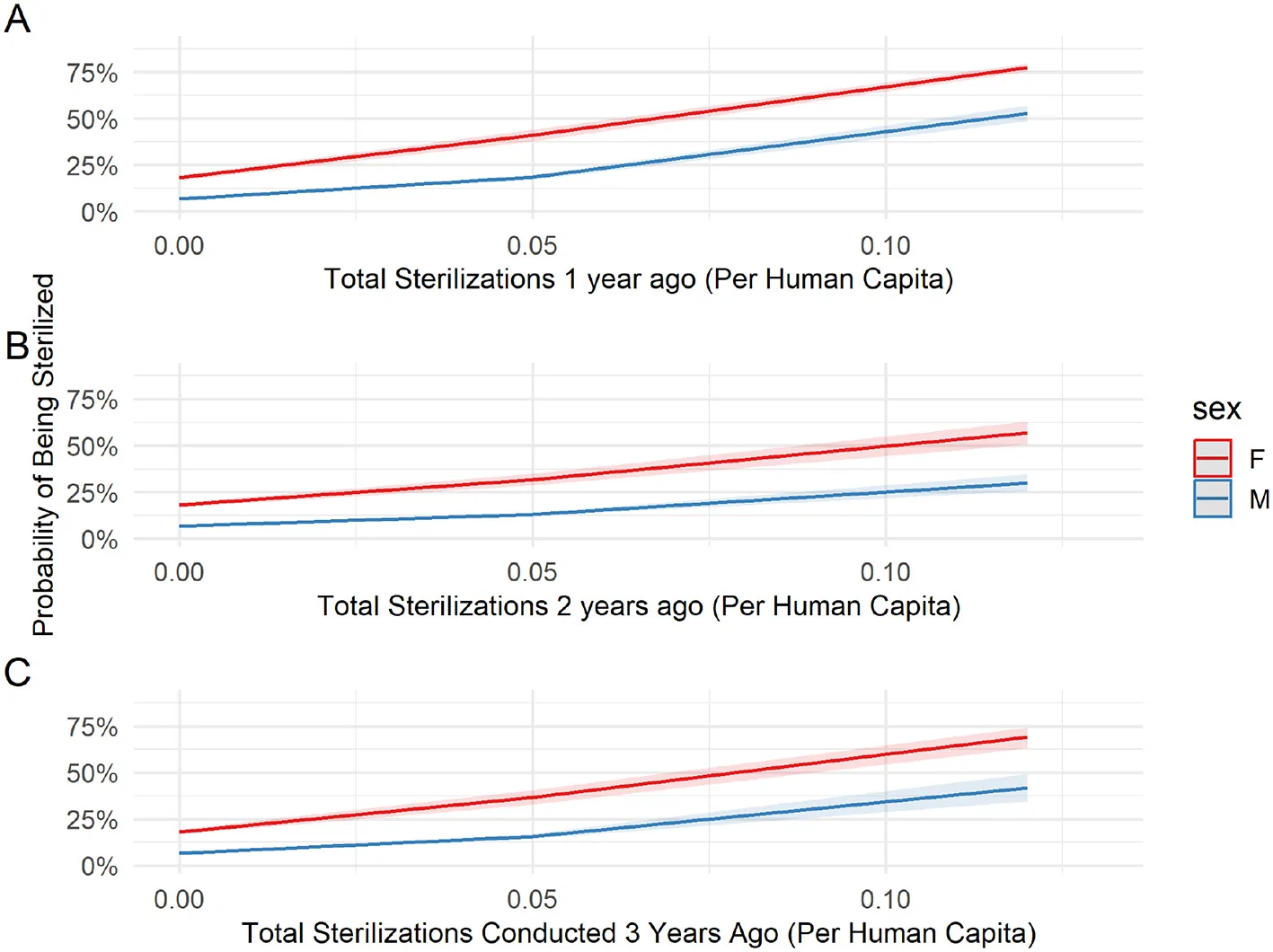
Predicted probability that a dog is sterilized by number of sterilizations conducted in past years and dog sex. The probability of a dog being sterilized based on the total number of sterilizations that had occurred 1 year **(A)**, 2 years **(B)**, and 3 years **(C)** before the transect date, dividing model based on sex (female is red and male is blue), if there were no sterilizations conducted in the other years. Shaded envelopes indicate 95% confidence intervals.

**Table 2:** The best-fitting statistical model examining associations between Jai Dog's sterilization program and dog sterilization status.

Parameter type	Variable	Estimate	Standard error	*Z* value	*P*-value
Fixed effects	Intercept	−1.5116	0.3168	−4.771	< 0.001
	Sterilization effort 3 years ago	19.2941	4.9665	3.885	< 0.001
	Sterilization effort 2 years ago	14.9054	3.9622	3.762	< 0.001
	Sterilization effort 1 year ago	22.9457	3.0088	7.626	< 0.001
	Sex (Male)	−1.1295	0.2440	−4.629	< 0.001
Random effect variance	Polygon	0.2534			

Effort was defined as the total sterilizations conducted 3, 2, and 1 year ago. Other models with alternative measures of effort are shown in the Supplementary materials.

The KAP survey reflects the sterilization status of owned dogs as reported by respondents. Owned dogs were defined as owned by the respondent, they may or may not be allowed to roam unsupervised. When defining the number of dogs they owned, respondents were asked to exclude any street dogs that they provided care for. The intervention district had a significantly higher sterilization rate of owned dogs than the control district (71 vs. 45%, *p* < 0.001, Figure 6b). Respondents from the intervention area were additionally significantly more likely to prefer humane dog population interventions. Management options such as rehoming, vaccination and return, sterilization and return, and CNVR were selected by 73.2% of respondents in the intervention and 50.7% of respondents in the control. The difference of 22.5 percentage points between control and intervention respondents was significant (95% CI 11.8–32.5; exploratory Pearson χ^2^(1) = 17.03, *p* < 0.001; Figure 6a). These findings describe an unadjusted between-district difference and should not be interpreted as an independent effect of Jai Dog's activities.

**Figure 6:**
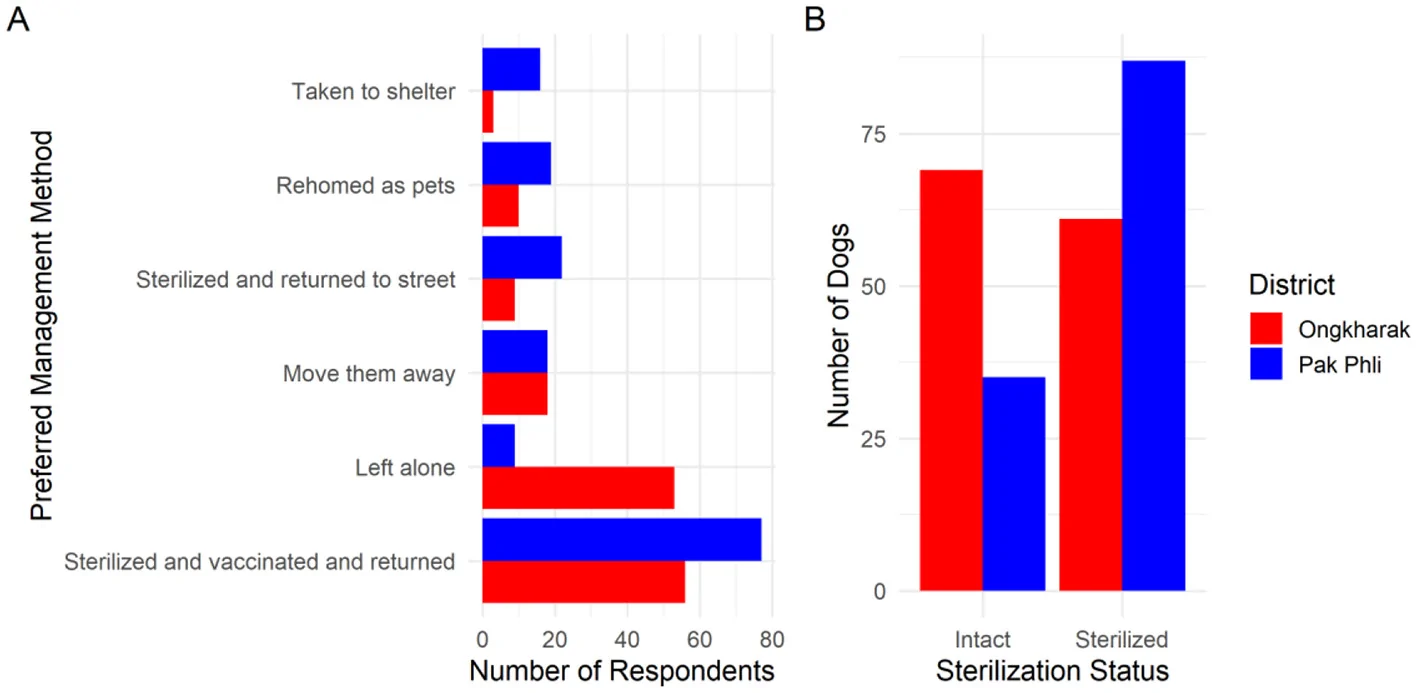
Respondents' attitudes and behavior toward DPM and dog sterilization. **(A)** Respondents' preferred method of managing the dog population. **(B)** Number of owned dogs that are sterilized by district. Red bars correspond to Pak Phli district where the CNVR intervention was carried out, while blue bars correspond to Ongkharak district which was the control area with no intervention.

### 3.3 Objective 3: lactating females and puppies

#### 3.3.1 Lactating females

No significant effects of our explanatory variables were found on the presence of lactating females.

#### 3.3.2 Puppies

We found a significant association between all measures of sterilization effort and the probability that a sighted a dog is a puppy only when the full dataset, with both days of observations, was used. When comparing by AIC, time since intervention was the best fitting sterilization measure (since intervention AIC:151.91, total effort AIC: 154.09, sterilization effort by year AIC: 152.03). Increasing time since the peak of the intervention was significantly associated with decreased probability that an observed dog was a puppy and the probability of being a puppy was significantly higher in Tha Chang (Table 3, Figure 7). The probability of an observed dog being a puppy was quite low pre-intervention, at 0.03 (95% CI 0.01–0.07) in Khok Kruat and 0.1 (95% CI 0.05–0.18) in Tha Chang. The estimated prevalence of puppies, while low, steadily declined in the years following the intervention (Figure 7), to 0.003 (95% CI 0.0005–0.02) in Khok Kruat and 0.01 (95% CI 0.003–0.04) in Tha Chang 3 years after the peak of the intervention. When this model was tested with day two data removed, time since intervention no longer showed significance on puppy prevalence (*p* = 0.06). There was an overall low prevalence of puppies within our sample. Of the 18 total puppies observed in Khok Kruat there were 2 puppies in 2021 and 3 in 2023, and in Tha Chang there were 10 puppies in 2023 and 3 in 2024. Due to the small number of puppies in our data further sensitivity analyses were conducted. In the original final model, time since intervention had an odds ratio of 0.474. When tested with firth logistic regression time since intervention had an odds ratio of 0.498. A leave one puppy out sensitivity analysis, where the effect of excluding one of the puppies and then refitting the model each time, resulted in an odds ratio range of 0.432–0.498, showing that results were not strongly influenced by any single puppy. Consistency in the odds ratio across the tests demonstrates the stability of the model.

**Table 3:** The best-fitting statistical models examining associations between Jai Dog's sterilization program and probability of a dog being a puppy.

		Time since intervention model				Annual sterilization effort			
Parameter type	Variable	Estimate	Std. error	*Z* value	*P*-value	Estimate	Std. error	*Z* value	*P*-value
Fixed effects	Intercept	−3.4688	0.4596	−7.548	< 0.001	−2.7383	0.2844	−9.630	< 0.001
	Time since intervention	−0.7466	0.2895	−2.579	0.0099				
	Sterilization effort 2 years ago					−48.4512	20.2055	−2.398	0.0165
	Subdistrict (Tha Chang)	1.2585	0.5599	2.248	0.0246				

Effort was defined as time since intervention and annual sterilization effort. Other models with alternative measures of effort are shown in the Supplementary materials.

**Figure 7:**
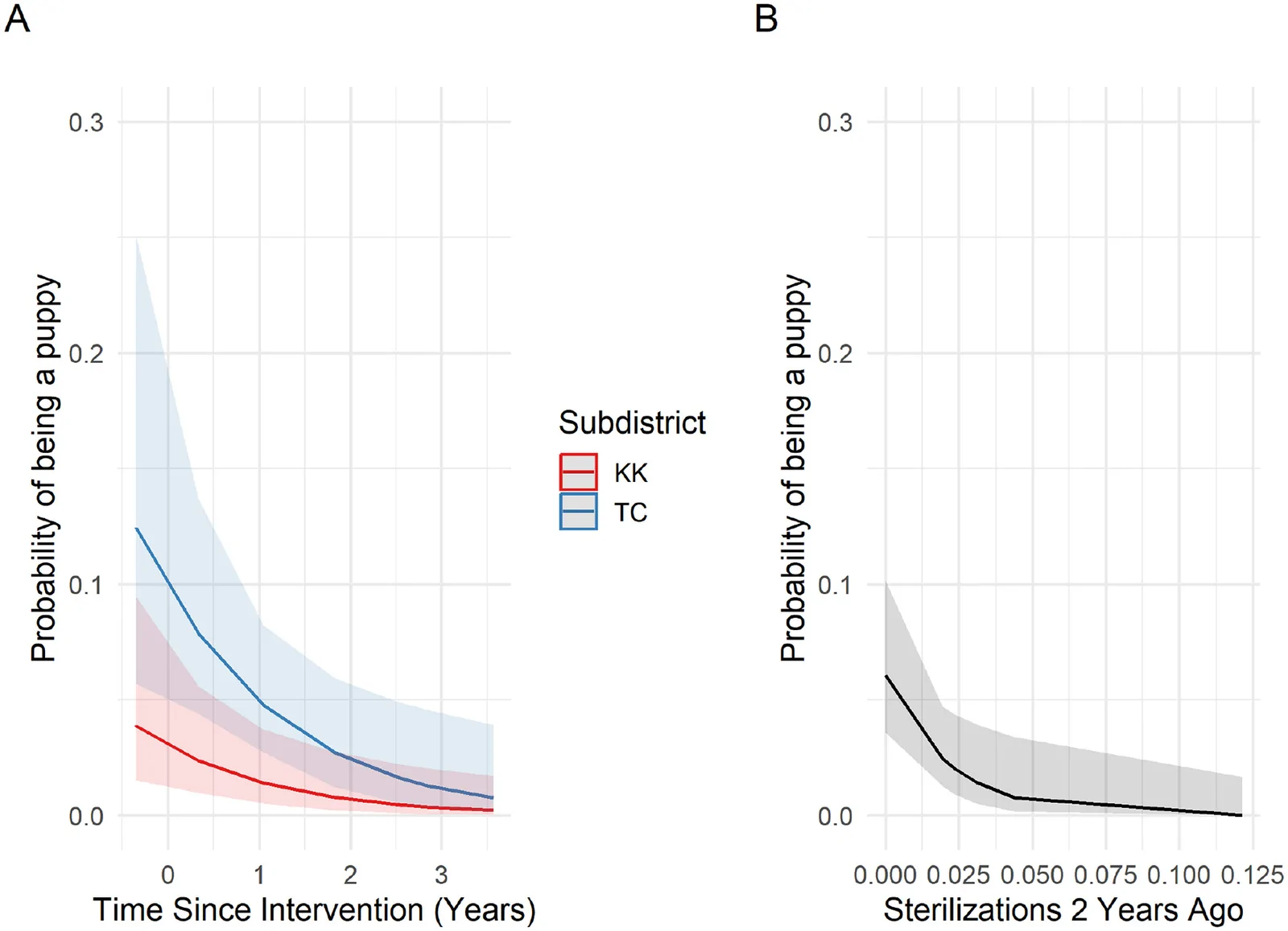
Probability that a dog is a puppy in relation to time since intervention. Decline in probability of observing a puppy in association with time **(A)** since the peak of the intervention, measured in years and by **(B)** annual sterilization effort 2 years before survey date. Lines demonstrate the model fit with the subdistrict of Khok Kruat colored red, and Tha Chang colored blue. Shaded envelopes indicate 95% confidence intervals.

### 3.4 Objective 4: welfare-related indicators

None of the measures of sterilization effort (time since intervention, total sterilization effort, or sterilization by year) had a significant effect on the probability of a dog being healthy. Our other explanatory variables of sex, age, ownership status, and sterilization status also had no detectable effect. The KAP survey included questions asking if respondents had encountered any problems related to seeing sick or injured street dogs, and only 2% of respondents indicated they had. No respondents indicated that they had provided medical care for a street dog in the last year. Respondents in the intervention district reported seeking more veterinary care for their owned dogs as compared to the control district (45%), but only in terms of higher numbers of sterilizations (71%) rather than general health care.

## 4 Discussion

### 4.1 Key findings

Our findings suggest that Jai Dog's DPM initiatives are associated with desired changes within the dog population that are meaningful enough to be reflected in citizens' perceptions. These changes were additionally sustained across several years after the peak of a one-time high-intensity sterilization clinic and a small number of ongoing sterilizations to maintain sterilization coverage, alongside other complementary DPM programming. First, Jai Dog's work was associated with a decrease in free-roaming dogs observed per km *via* transect counts, consistent with the KAP survey reports of fewer perceived dogs in areas with DPM. Moreover, KAP survey respondents also reported a decreased incidence of nuisance dog behaviors and dog bites in areas where Jai Dog worked. Additionally, Jai Dog's activities were associated with increased likelihood of observed dogs being sterilized, supported by the KAP survey, with respondents from the intervention area reporting higher sterilization rates within their owned dogs. There are indications that Jai Dog's work also positively shaped attitudes toward DPM, with KAP survey respondents who lived in areas where the organization was present reporting greater support for humane DPM interventions such as CNVR. Finally, we found a slight significant effect on the presence of puppies in the population associated with Jai Dog's DPM effort. While this significance was not present when tested with day two data removed, it does suggest a statistical trend is present. A decline in puppies serves as an important indicator that fewer litters are being born, therefore decreasing the population. Furthermore, the results of the transects were generally consistent with or without 2 days of data, indicating that one day of observations in each polygon each year is sufficient for identifying desired changes in the dog population. Combined, these measures demonstrate that Jai Dog's work results in changes in the larger dog population, both free-roaming and owned dogs.

### 4.2 Broader context

Jai Dog's work is an example of a comprehensive DPM programme that extends beyond CNVR of stray free-roaming dogs but also addresses the owned dog population. Community members' acceptance of CNVR, as highlighted in the KAP survey results, suggests support for Jai Dog's initiatives. Jai Dog's sterilization campaign structure included a onetime mobile clinic on location in subdistricts, providing sterilization services to owned dogs as well community-owned and stray dogs, there was also ongoing opportunity for dog owners and the Jai Dog catchers to bring dogs to the nearby permanent clinic at Jai Dog's headquarters. The large onetime pulse of sterilizations followed by ongoing maintenance through a trickle effect of sterilizations suggests that alongside Jai Dog's other programming, dogs observed per km and the prevalence of puppies were sufficiently reduced with sterilization coverage controlled and no rebound observed in the subsequent years of transect data. While these results do not establish causality, they provide coherent evidence supporting the role of sterilization in improving animal welfare and human-dog coexistence. These findings confirm previous reports that sterilization based DPM campaigns are positively associated with increases in sterilization coverage and decreases in dog population size and puppies observed (14, 18, 31). Our analysis of sterilization coverage showed that male dogs were significantly less likely to be sterilized than females. Other studies in the Thai context have found that many dog owners believe male dogs do not need to be sterilized (21, 32), and thus may have been less likely to bring their male dogs to sterilization clinics. The KAP survey analysis further aligns with existing findings that mass dog sterilization is associated with changes in community member's perceptions of dog numbers and nuisance behaviors (23, 31, 32). Our analysis is unique in that it demonstrates impact with a comparatively low level of DPM effort; most literature reports changes in dog counts and sterilization coverage after 2–5 years of various intensities of regular CNVR effort (10, 13, 33).

One important aspect of Jai Dog's sterilization campaigns is that their intervention encompasses all segments of the dog population. There are four categories of dogs identified by Jai Dog: stray (feral), community-owned (roaming), owned (roaming), and owned (confined). A very small percentage of dogs sterilized by the mobile and permanent clinics were strays, the majority were owned (mostly roaming and some confined) or community-owned (roaming). Each segment of the dog population can contribute to increases in dog numbers, through abandonment of intact dogs and their offspring or allowing intact dogs to free roam. Therefore, studies have found that reducing multiple sections of the dog population through sterilization alongside interventions such as responsible ownership education is most effective in managing dog numbers (13, 34, 35). Providing owners with access to sterilization services to control reproduction in their owned dogs is particularly key in reducing the dog population as it reduces potential for abandonment of unwanted litters of puppies (13, 34). The movement of intact dogs into a population makes it difficult to maintain sterilization coverage and reverses the effects of CNVR (34). Jai Dog's program encompasses these different segments of the dog population to establish comprehensive sterilization coverage across all potential sources of dog population growth, as demonstrated in the high levels of sterilization coverage identified in the dogs observed on the transects and respondents' reports of owned dogs.

Running the models with and without second day transect data demonstrated that had the transects only been completed once in each polygon each year, there still would have been sufficient data to observe a significant decline in dogs observed per km and increases in sterilization coverage. This is important operationally as it shows that to identify changes in the indicators explored in this study, one day of transect data is sufficient, saving the associated time and costs of conducting further data collection. However, this finding does not consider other analyses which may use resights, such as dog population size estimation using mark-resight techniques. Therefore, when the objective is to solely measure changes within the dog population, one day of transect data may be sufficient.

### 4.3 Strengths and limitations

The greatest strength of our study is that it offers a multi-year examination of dog populations both before and after DPM intervention as well as a comparison of intervention and control areas. It further combines the methods of transect counts and KAP surveys, to explore quantitative measures of changes in observations of free-roaming dogs and whether they are of significant magnitude to be noticed and meaningful to local residents. In this study the KAP component was retained as an exploratory, unadjusted comparison intended to provide descriptive context alongside the longitudinal transect findings. The KAP survey responses suggest that between-district differences were directionally consistent with transect findings. However, further multivariable analysis is required to fully take into account the effects of other predictors and district level differences on respondents' perceptions. Further qualitative work (such as in-depth interviews) could build upon KAP survey results for deeper understanding on how roaming dogs, and Jai Dog's DPM work, has impacted the lived realities of local communities.

The transect data was focused on the subdistricts where Jai Dog has been active the longest, thus providing the most years of data. While using these two subdistricts gave us the maximum number of transects possible, there were unexplained differences in the dog counts between the subdistricts at their baseline, with Tha Chang having a lower count of dogs per km but higher puppy prevalence than Khok Kruat pre-intervention. There may be factors at play that we are not fully aware of that explain this variation. One key difference is that the mobile clinic in Tha Chang took place after Jai Dog had already been working in neighboring areas, and established their permanent clinic nearby, meaning dogs in the Tha Chang were already subject to a low level of DPM effort (Figure 2). Khok Kruat additionally has a higher human density (Khok Kruat: 231 people per square km; Tha Chang: 198 people per square km). The difference in baseline dog population numbers does not change the demonstrated impact of Jai Dog's intervention, consistent across both subdistricts, however it does raise concerns over accurately comparing dog numbers across regions such as in the KAP survey. The one-time KAP survey suggests that respondents in the intervention district reported lower numbers of dogs than those in the control. However, there may be overall differences in dog numbers in these two districts, regardless of Jai Dog's intervention. The variation in baseline dog numbers in the two subdistricts may also limit similarity between the dog: human ratios across the study areas, affecting the accuracy of the sterilizations per human capita variable used throughout our analysis.

Our analysis did not detect significant changes in numbers of lactating females and dog health status, two key DPM impact indicators identified in other research (8, 14) and prioritized outcomes by the Jai Dog team. Both these groups made up a small percentage of our dataset (lactating females *n* = 9, 2%, sick dogs *n* = 39, 7%), hindering the ability to identify a significant difference. To detect changes in these indicators, further data collection and analysis would be required. Although there is little research in the Thai context regarding the seasonality of dog births, other studies suggest that there may be patterns related to birth rates and time of year (12, 36). Hence there may be times of the year when the chance of observing lactating females and puppies would be higher. We did not identify seasonality in pregnancy in our focus area, but further data collection may reveal a pattern.

Similarly, continued monitoring of dog health and community practices could reveal temporal trends in the free-roaming dog population and with Jai Dog's clinical records. This study employed a simple binary measure of healthy or unhealthy, based on visual assessment of dogs from a distance during the transect survey. This resulted in an indicator of health status that only recognized dogs that were visibly ill as “unhealthy”. There were additionally very few dogs with thin body condition scores, or poor skin condition, at baseline. Gaining a more complete understanding of the multiple facets of dog welfare in this context is complex. While the KAP survey did not record respondent's perceptions of dog health, it did show that higher numbers of owned dogs in the intervention area are sterilized, representing greater veterinary care from either Jai Dog or other local veterinary services being sought. If Jai Dog wished to further prioritize the assessment of dog health implications, more comprehensive welfare-focused evaluation could be conducted examining physical and behavioral indicators of dog welfare and people's practices and perceptions surrounding dog care (8).

This research does not directly focus on rabies transmission and vaccination. Rabies incidents in our province of study are rare, therefore the impact of Jai's sterilization effort on rabies transmission could not be analyzed. However, as rabies is a major concern related to DPM globally, our study provides transferable insights regarding dog population dynamics. While sterilization does not directly impact rabies transmission (37), it reduces population turnover, helping maintain vaccination coverage, as evidenced by the small and declining number of puppies observed in areas where Jai Dog works (5, 38). Reaching vaccination rates of upwards of 70% remains the best way to curb rabies transmission (39). All dogs that were sterilized at the CNVR clinics, were also ear notched and vaccinated with DHPPi+LR vaccines (full vaccination protocol can be found in supplementary materials 4). The exact protective immunity conferred by this vaccination cannot be confirmed as the immune response may vary by individual and be impacted by stress related factors associated with surgical sterilization (40). Therefore, transect counts that identify sterilized dogs based on ear notching, can also provide a measures of the number of observed dogs that had received at least one vaccination dose (Figure 5). This measure cannot make wider inferences on vaccination coverage as it does not take into account vaccinations conducted by actors other than Jai Dog or the number of vaccinated dogs that were not observed via the transects.

### 4.4 Recommendations and conclusions

We have demonstrated that both transect and KAP surveys are effective in measuring the impact of DPM. The findings of both evaluation methods were complementary and directionally consistent in evidencing favorable dog-population patterns and community experiences in areas where Jai Dog implemented its DPM programme. This work offers a strong foundation to show the current impact of Jai Dog's activities and the potential for future success through continued use of their monitoring approaches. Our findings contribute to filling the gap in existing research evaluating the success of DPM in Thailand outside of Bangkok. Furthermore, it demonstrates that Jai Dog's work has been associated with obtaining desired changes in the dog population and that their survey methods are valuable tools in quantifying these impacts. We conclude that effective monitoring of sterilization programmes shows promising impact in terms of reducing dogs observed per km and population turnover, providing benefits to both humans and dogs.

## Data Availability

All data and code used in this analysis is available at: https://github.com/BronteS003/Jai_Dog.

## Supplementary material

Supplementary material 1 Additional Models.

Supplementary material 2 Day 2 Comparison.

Supplementary material 3 Statement on Use of Generative AI.

Supplementary material 4 CNVR Protocols.
