# Supplementary material for: The impacts of dog sterilization campaigns in Thailand: understanding changes in dog population dynamics
Source: Front Vet Sci. 2026 Oct 27;13:1926523. doi: 10.3389/fvets.2026.1926523 (PMC13747784; doi:10.3389/fvets.2026.1926523)
Supplement: Supplementary file 1 [file Supplementary_file_1.docx]

# Supplementary Materials 1: Additional Models

The impact of Jai Dog’s work was quantified across three measures of effort: time since the peak of the intervention, total sterilization effort up to the date of the survey, and annual sterilization effort (4, 3, 2, and 1 year before the survey date). For each objective, models were fitted with each measure of sterilization effort and the final models compared using AIC. The model with the best AIC is presented in the paper, however the other fitted models are included here in the supplementary materials.

### Objective 1: Free-roaming Dogs Observed per Km

Full Model 1:

$$\log\left( f_{t} \right)= \beta_{0}+\beta_{e}e_{t}+ a_{subdistrict(t)}+ t_{transport(t)}+ d_{day(i)}+ u_{p}+ v_{sp}+\log(l_{p(t)})$$

$$u_{p}\sim N\left( 0,\sigma_{u}^{2} \right)$$

$$v_{sp}\sim N(0,\sigma_{v}^{2})$$

*f* = fitted mean count

$t$ = transect

$\beta_{0}$ = intercept

$e$ = sterilization effort

$a$ *=* subdistrict (Khok Kruat or Tha Chang)

$t$ = transport (factor: foot, bicycle, two-wheeled vehicle, four-wheeled vehicle)

$d$ = day (factor: day 1, day 2)

$u_{p}$ = random effect of polygon (7 levels: 3 in Khok Kruat and 4 in Tha Chang)

$v_{sp}$ = random effect of survey id nested in polygon (7 levels: 3 in Khok Kruat and 4 in Tha Chang)

$l_{p}$ = transect length in polygon *p*

#### All Measures of Effort

**Table 1. Comparison of Dogs Observed per Km Models with Different Measures of Sterilization Effort**

| **Model** | **AIC** |
| --- | --- |
| Model 1 – Time Since Intervention | 265.07 |
| Model 1 – Total Sterilization Effort | 265.92 |
| Model 1 – Sterilization Effort by Year | 270.38 |

#### Time Since Intervention

Final model – Time Since Intervention

$$\log\left( f_{t} \right)= \beta_{0}+\beta_{e}e_{t}+ a_{subdistrict (t)}+ u_{p}+ v_{sp}+\log(l_{p(t)})$$

$e$ = years since peak of intervention

**Table 1.1. Statistical Model Examining Associations Between Jai Dog’s Time Since Intervention and Changes to Dogs Observed per Km**

|  | **Variable** | **Estimate** | **Standard Error** | **Z value** | **P-value** |
| --- | --- | --- | --- | --- | --- |
| **Fixed Effects** | Intercept | 2.3366 | 0.2855 | 8.183 | <0.001 |
|  | Time Since Intervention | -0.1712 | 0.0468 | -3.657 | 0.0003 |
|  | Subdistrict (Tha Chang) | -1.2553 | 0.3715 | -3.379 | 0.0007 |
| **Random Effect Variance** | Polygon | 0.2100 |  |  |  |
|  | Survey:Polygon | 0.0329 |  |  |  |

#### Total Sterilization Effort

Final model – Total Sterilization Effort:

$$\log\left( f_{t} \right)= \beta_{0}+\beta_{e}e_{t}+ a_{subdistrict (t)}+ u_{p}+ v_{sp}+\log(l_{p(t)})$$

$e$ = measure of total sterilization

**Table 1.2. Statistical Model Examining Associations Between Jai Dog’s Total Sterilization Effort and Changes to Dogs Observed per Km**

|  | **Variable** | **Estimate** | **Standard Error** | **Z value** | **P-value** |
| --- | --- | --- | --- | --- | --- |
| **Fixed Effects** | Intercept | 2.3526 | 0.2871 | 8.195 | <0.001 |
|  | Total sterilisation effort | -2.5356 | 0.7262 | -3.491 | 0.0005 |
|  | Subdistrict (Tha Chang) | -1.0850 | 0.3689 | -2.941 | 0.0033 |
| **Random Effect Variance** | Polygon | 0.2083 |  |  |  |
|  | Survey:Polygon | 0.0356 |  |  |  |

#### Sterilization Effort by Year

Final model – Sterilization Effort by Year:

$$\log\left( f_{t} \right)= \beta_{0}+\beta_{A}A_{t}+ a_{subdistrict (t)}+ u_{p}+ v_{sp}+\log(l_{p(t)})$$

A = measure of sterilization effort 1 year ago

**Table 1.3. Statistical Model Examining Associations Between Jai Dog’s Annual Sterilization Effort and Changes to Dogs Observed per Km**

|  | **Variable** | **Estimate** | **Standard Error** | **Z value** | **P-value** |
| --- | --- | --- | --- | --- | --- |
| **Fixed Effects** | Intercept | 2.2437 | 0.2820 | 7.956 | <0.001 |
|  | Sterilization Effort 1 Year Ago | -3.8342 | 1.6187 | -2.369 | 0.0179 |
|  | Subdistrict (Tha Chang) | -1.0047 | 0.3659 | -2.746 | 0.0060 |
| **Random Effect Variance** | Polygon | 0.19518 |  |  |  |
|  | Survey:Polygon | 0.06064 |  |  |  |

### Objective 2: Sterilized Dogs

Full Model 2:

$$\log\left( \frac{P_{i}}{1-P_{i}} \right)= \beta_{0}+\beta_{e}e_{i}+ a_{subdistrict (i)}+ o_{ownership(i)}+ s_{sex(i)}+ u_{p(i)}$$

$P$ = probability of a dog being sterilized

$i$ = individual dog observation

$o$ = ownership status (factor: owned, unowned)

$s$ = sex (factor: female, male)

#### All Measures of Effort

**Table 2. Comparison of Sterilization Status Models with Different Measures of Sterilization Effort**

| **Model** | **AIC** |
| --- | --- |
| Model 2 – Time Since Intervention | 504.8525 |
| Model 2 – Total Sterilization Effort | 483.1821 |
| Model 2 – Sterilization Effort by Year | 475.9618 |

#### Time Since Peak of Intervention

Final model – Time Since Intervention

$$\log\left( \frac{P_{i}}{1-P_{i}} \right)= \beta_{0}+\beta_{e}e_{i}+ {o_{ownership(i)}+ s}_{sex(i)}+ u_{p(i)}$$

**Table 2.1. Statistical Model Examining Associations Between Jai Dog’s Sterilization Effort and the Likelihood of a Dog Being Sterilized by Time Since the Peak of Intervention**

|  | **Variable** | **Estimate** | **Standard Error** | **Z value** | **P-value** |
| --- | --- | --- | --- | --- | --- |
| **Fixed Effects** | Intercept | -1.2695 | 0.2921 | -4.346 | <0.001 |
|  | Time Since Intervention | 1.0422 | 0.1060 | 9.835 | <0.001 |
|  | Owned (Yes) | 0.4720 | 0.2309 | 2.044 | 0.041 |
|  | Sex (Male) | -1.0100 | 0.2296 | -4.399 | <0.001 |
| **Random Effect Variance** | Polygon | 0.1534 |  |  |  |

#### Total Sterilization Effort

Final model – Total Sterilization Effort

$$\log\left( \frac{P_{i}}{1-P_{i}} \right)= \beta_{0}+\beta_{e}e_{i}+ s_{sex(i)}+ u_{p(i)}$$

**Table 2.2. Statistical Model Examining Associations Between Jai Dog’s Sterilization Effort and Likelihood of a Dog Being Sterilized by Total Sterilization Effort**

|  | **Variable** | **Estimate** | **Standard Error** | **Z value** | **P-value** |
| --- | --- | --- | --- | --- | --- |
| **Fixed Effects** | Intercept | -1.3164 | 0.2934 | -4.486 | <0.001 |
|  | Total Sterilization Effort | 17.2198 | 1.6329 | 10.546 | <0.001 |
|  | Sex (Male) | -1.0717 | 0.2382 | -4.499 | <0.001 |
| **Random Effect Variance** | Polygon | 0.2209 |  |  |  |

#### Sterilization Effort by Year

Final model – Sterilization effort by year

$$\log\left( \frac{P_{i}}{1-P_{i}} \right)= \beta_{0}+\beta_{A}A_{i}+ \beta_{B}B_{i}+ \beta_{C}C_{i}+ s_{sex(i)}+ u_{p(i)}$$

**Table 2.3. Statistical Model Examining Associations Between Jai Dog’s Sterilization Effort and Likelihood of a Dog Being Sterilized by Year**

|  | **Variable** | **Estimate** | **Standard Error** | **Z value** | **P-value** |
| --- | --- | --- | --- | --- | --- |
| **Fixed Effects** | Intercept | -1.5116 | 0.3168 | -4.771 | <0.001 |
|  | Sterilization Effort 3 Years Ago | 19.2941 | 4.9665 | 3.885 | <0.001 |
|  | Sterilization Effort 2 Years Ago | 14.9054 | 3.9622 | 3.762 | <0.001 |
|  | Sterilization Effort 1 Year Ago | 22.9457 | 3.0088 | 7.626 | <0.001 |
|  | Subdistrict (Tha Chang) | -1.1295 | 0.2440 | -4.629 | <0.001 |
| **Random Effect Variance** | Polygon | 0.2534 |  |  |  |

### Objective 3.1 & 3.2: Presence of Lactating Females & Puppies

Full Model 3.1 and 3.2:

$$\log\left( \frac{P_{i}}{1-P_{i}} \right)= \beta_{0}+\beta_{e}e_{i}+ a_{subdistrict(i)}+ o_{ownership(i)}$$

### Objective 3.2: Presence of Puppies

#### All Measures of Effort

**Table 3. Comparison of Sterilization Status Models with Different Measures of Sterilization Effort**

| **Model** | **AIC** |
| --- | --- |
| Model 3.2 – Time Since Intervention | 151.9149 |
| Model 3.2 – Total Sterilization Effort | 154.0861 |
| Model 3.2 – Sterilization Effort by Year | 152.0328 |

#### Time Since Intervention

Final model – Time Since Intervention

$$\log\left( \frac{P_{i}}{1-P_{i}} \right)= \beta_{0}+\beta_{e}e_{i}+ a_{subdistrict (i)}$$

**Table 3.1. Statistical Model Examining Associations Between Jai Dog’s Sterilization Effort and Likelihood of a Dog Being a Puppy by Time Since Intervention**

| **Variable** | **Estimate** | **Standard**  **Error** | **Z value** | **P-value** |
| --- | --- | --- | --- | --- |
| Intercept | -3.4688 | 0.4596 | -7.548 | <0.001 |
| Time Since Intervention | -0.7466 | 0.2895 | -2.579 | 0.0099 |
| Subdistrict (Tha Chang) | 1.2585 | 0.5599 | 2.248 | 0.0246 |

#### Total Sterilization Effort

Final model – Total Sterilization Effort

$$\log\left( \frac{P_{i}}{1-P_{i}} \right)= \beta_{0}+\beta_{e}e_{i}$$

*P* = probability of a dog being a puppy

**Table 3.2. Statistical Model Examining Associations Between Jai Dog’s Sterilization Effort and Likelihood of a Dog Being a Puppy by Total Sterilization Effort**

| **Variable** | **Estimate** | **Standard**  **Error** | **Z value** | **P-value** |
| --- | --- | --- | --- | --- |
| Intercept | -2.7121 | 0.2952 | -9.187 | <0.001 |
| Effort All Time | -9.7423 | 3.7175 | -2.621 | 0.0088 |

#### Sterilization Effort by Year

Final model – Sterilization Effort by Year:

$$\log\left( \frac{P_{i}}{1-P_{i}} \right)= \beta_{0}+ \beta_{B}B_{i}+a_{subdistrict (i)}$$

**Table 3.3. Statistical Model Examining Associations Between Jai Dog’s Sterilization Effort and Likelihood of a Dog Being a Puppy by Annual Sterilization Effort**

| **Variable** | **Estimate** | **Standard**  **Error** | **Z value** | **P-value** |
| --- | --- | --- | --- | --- |
| Intercept | -2.7383 | 0.2844 | -9.630 | <0.001 |
| Sterilization Effort 2 Years Ago | -48.4512 | 20.2055 | -2.398 | 0.0165 |

### Objective 4: Presence of Sick Dogs

Full Model 4:

$$\log\left( \frac{P_{i}}{1-P_{i}} \right)= \beta_{0}+\beta_{e}e_{i}+ a_{subdistrict(i)}+ o_{ownership(i)}+ s_{sex(i)}+ g_{age(i)}+ n_{neutered(i)}+ u_{p(i)}$$

$g$ = age (factor: puppy, adult)

*n* = sterilization status (factor: sterilized, intact)
