# Supplementary material for: The impacts of dog sterilization campaigns in Thailand: understanding changes in dog population dynamics
Source: Front Vet Sci. 2026 Oct 27;13:1926523. doi: 10.3389/fvets.2026.1926523 (PMC13747784; doi:10.3389/fvets.2026.1926523)
Supplement: Supplementary file 2 [file Supplementary_file_2.docx]

# Supplementary Materials 2: Day Comparison

Identifying whether both days of transect data are required to observe desired changes in the dog population.

The statistical models in the current analysis included two days of surveying, where resighted dogs were retained in model 1 and removed in models 2-4 to avoid duplication of the same dog observations. Each model was re-run, removing all data from day 2 to determine if the additional day of surveying was important in identifying the observed significant declines in dog density (model 1) and puppies (model 3.2), and increase in sterilization coverage (model 2).

### Model 1 – Dog Density

The results of the dog density model with and without day 2 data were near identical. Both models showed that all measures of sterilization effort were associated with significant decreases in dog density. However, the comparison of AICs determining that the best fitting measures of sterilization effort in both instances were time since the peak of the intervention and total effort (table 1, table 2). With and without day 2 data, time since intervention and total sterilization effort had a high level of significance (p <0.001).

**Table 1. Statistical summary of original dog density model**

|  |  | **Time since intervention model** | | | | **Total sterilisation effort model** | | | |
| --- | --- | --- | --- | --- | --- | --- | --- | --- | --- |
|  | **Variable** | **Estimate** | **Standard Error** | **Z value** | **P-value** | **Estimate** | **Standard Error** | **Z value** | **P-value** |
| **Fixed Effects** | Intercept | 2.3366 | 0.2855 | 8.183 | <0.001 | 2.3526 | 0.2871 | 8.195 | <0.001 |
|  | Time Since Intervention | -0.1712 | 0.0468 | -3.657 | 0.0003 |  |  |  |  |
|  | Total sterilisation effort |  |  |  |  | -2.5356 | 0.7262 | -3.491 | 0.0005 |
|  | Subdistrict (Tha Chang) | -1.2553 | 0.3715 | -3.379 | 0.0007 | -1.0850 | 0.3689 | -2.941 | 0.0033 |
| **Random Effect Variance** | Polygon | 0.2100 |  |  |  | 0.2083 |  |  |  |
|  | Survey:  Polygon | 0.0329 |  |  |  | 0.0356 |  |  |  |

**Figure 1. Changes in dog density in relation to time since intervention and total sterilization effort with full dataset.** A) Decline in dogs sighted in association with time since the peak of the intervention, measured in years. B) Decline in dogs sighted in association with the total number of sterilizations conducted up to transect date. Lines demonstrate the model fit with the subdistrict of Khok Kruat coloured red, and Tha Chang coloured blue. Points mark the average number of dogs observed per transect, while lines and shaded envelopes indicate 95% confidence intervals.

**Table 2. Statistical summary of dog density model with day 2 removed**

|  |  | **Time since intervention model** | | | | **Total sterilisation effort model** | | | |
| --- | --- | --- | --- | --- | --- | --- | --- | --- | --- |
|  | **Variable** | **Estimate** | **Std.**  **Error** | **Z value** | **P-value** | **Estimate** | **Std.**  **Error** | **Z value** | **P-value** |
| **Fixed Effects** | Intercept | 2.3221 | 0.3366 | 6.899 | <0.001 | 2.3293 | 0.3378 | 6.895 | <0.001 |
|  | Time Since Intervention | -0.1555 | 0.0402 | -3.869 | 0.0001 |  |  |  |  |
|  | Total sterilisation effort |  |  |  |  | -2.2873 | 0.6429 | -3.558 | 0.0004 |
|  | Subdistrict (Tha Chang) | -1.2605 | 0.4388 | -2.873 | 0.0041 | -1.1026 | 0.4362 | -2.528 | 0.0115 |
| **Random Effect Variance** | Polygon | 0.3011 |  |  |  | 0.2986 |  |  |  |
|  | Survey:  Polygon | 0.0025 |  |  |  | 0.0058 |  |  |  |

**Figure 2. Changes in dog density in relation to time since intervention and total sterilization effort with day 2 data removed.**

### Model 2 – Sterilization Status

The results of the sterilization status model without day 2 data, were similar to the original model. Both models found all measures of sterilization effort to be significant. For both models the best fitting measure of effort, through the comparison of AIC, was sterilization effort by year. While sterilization effort 1 year ago remains highly significant across all models ( p <0.001), the significance of the 3 and 2 years ago variable declines slightly (p <0.01) with the removal of day 2 data.

**Table 3. Statistical summary of original sterilization status model**

|  | **Variable** | **Estimate** | **Standard Error** | **Z value** | **P-value** |
| --- | --- | --- | --- | --- | --- |
| **Fixed Effects** | Intercept | -1.5116 | 0.3168 | -4.771 | <0.001 |
|  | Sterilization Effort 3 Years Ago | 19.2941 | 4.9665 | 3.885 | <0.001 |
|  | Sterilization Effort 2 Years Ago | 14.9054 | 3.9622 | 3.762 | <0.001 |
|  | Sterilization Effort 1 Year Ago | 22.9457 | 3.0088 | 7.626 | <0.001 |
|  | Sex (Male) | -1.1295 | 0.2440 | -4.629 | <0.001 |
| **Random Effect Variance** | Polygon | 0.2534 |  |  |  |

**Figure 3. Predicted probability that a dog is neutered by number of sterilizations conducted in past years and dog sex with the full data set.** The probability of a dog being sterilized based on the total number of sterilizations that had occurred 1 year (A), 2 years (B), and 3 years (C) before the transect date, dividing model based on sex (female is red and male is blue) if there was no sterilizations conducted in the other years. Shaded envelopes indicate 95% confidence intervals.

**Table 4. Statistical summary of sterilization status model with day 2 data removed**

|  | **Variable** | **Estimate** | **Standard Error** | **Z value** | **P-value** |
| --- | --- | --- | --- | --- | --- |
| **Fixed Effects** | Intercept | -1.1978 | 0.3429 | -3.493 | 0.0005 |
|  | Sterilization Effort 3 Years Ago | 18.6164 | 6.0483 | 3.078 | 0.0021 |
|  | Sterilization Effort 2 Years Ago | 13.0637 | 4.2556 | 3.070 | 0.0021 |
|  | Sterilization Effort 1 Year Ago | 23.8496 | 3.6499 | 6.534 | <0.001 |
|  | Sex (Male) | -1.3365 | 0.2876 | -4.629 | <0.001 |
| **Random Effect Variance** | Polygon | 0.222 |  |  |  |

**Figure 4. Predicted probability that a dog is neutered by number of sterilizations conducted in past years and dog sex with day 2 data removed.**

### Model 3.2 – Puppy Prevalence

The probability of observing a puppy was greatly affected by removing day two data. Without the second day of data no significant effects were observed between sterilization effort and puppy prevalence.

**Table 5. Statistical summary of original puppy prevalence model**

|  |  | **Time since intervention model** | | | | **Annual Sterilization Effort** | | | |
| --- | --- | --- | --- | --- | --- | --- | --- | --- | --- |
|  | **Variable** | **Estimate** | **Std.**  **Error** | **Z value** | **P-value** | **Estimate** | **Std.**  **Error** | **Z value** | **P-value** |
| **Fixed Effects** | Intercept | -3.4688 | 0.4596 | -7.548 | <0.001 | -2.7383 | 0.2844 | -9.630 | <0.001 |
|  | Time Since Intervention | -0.7466 | 0.2895 | -2.579 | 0.0099 |  |  |  |  |
|  | Sterilization Effort 2 Years Ago |  |  |  |  | -48.4512 | 20.2055 | -2.398 | 0.0165 |
|  | Subdistrict (Tha Chang) | 1.2585 | 0.5599 | 2.248 | 0.0246 |  |  |  |  |

**Figure 5. Probability that a Dog is a Puppy in relation to time since intervention with full data set.** Decline in probability of observing a puppy in association with (A) time since the peak of the intervention, measured in years, and annual sterilization effort two years ago (B). Lines demonstrate the model fit, with panel A including the subdistrict of Khok Kruat coloured red, and Tha Chang coloured blue. Shaded envelopes indicate 95% confidence intervals.

**Table 6. Statistical summary of original puppy prevalence model with day 2 removed**

|  |  | **Time since intervention model** | | | | **Annual Sterilization Effort** | | | |
| --- | --- | --- | --- | --- | --- | --- | --- | --- | --- |
|  | **Variable** | **Estimate** | **Std.**  **Error** | **Z value** | **P-value** | **Estimate** | **Std.**  **Error** | **Z value** | **P-value** |
| **Fixed Effects** | Intercept | -4.1291 | 0.7223 | -5.716 | <0.001 | -4.1441 | 0.7453 | -5.560 | <0.001 |
|  | Time Since Intervention | -0.5883 | 0.3169 | -1.856 | 0.0634 |  |  |  |  |
|  | Sterilization Effort 2 Years Ago |  |  |  |  | -28.3244 | 16.8737 | -1.679 | 0.0932 |
|  | Subdistrict (Tha Chang) | 1.8451 | 0.8030 | 2.298 | 0.0216 | 1.7151 | 0.7869 | 2.180 | 0.0293 |

**Figure 6. Probability that a Dog is a Puppy in relation to time since intervention with day 2 data removed.**
