# Supplementary material for: The impacts of dog sterilization campaigns in Thailand: understanding changes in dog population dynamics
Source: Front Vet Sci. 2026 Oct 27;13:1926523. doi: 10.3389/fvets.2026.1926523 (PMC13747784; doi:10.3389/fvets.2026.1926523)
Supplement: Supplementary file 3 [file Supplementary_file_3.docx]

# Supplementary Materials 3: Generative AI Use Acknowledgement

The AI tool ChatGPT (GPT-5.5) was used for revision, troubleshooting and proofing

The “Materials and Methods” and “Results” sections of this paper contain results influenced by feedback received from ChatGPT. All feedback received from ChatGPT was reviewed and assessed for its accuracy. A full break down of each task and example prompts are included below.

Tasks used for:

- Revision: Enhancing understanding of R, GLMMs, and statistical tests.

Example prompts and outputs:

- Troubleshooting: Troubleshooting R error messages or unknown code.

Example prompts and outputs:

- Proofing: Checking the interpretation of tests.

Example prompts and outputs:
