# Supplementary material for: The impacts of dog sterilization campaigns in Thailand: understanding changes in dog population dynamics
Source: Front Vet Sci. 2026 Oct 27;13:1926523. doi: 10.3389/fvets.2026.1926523 (PMC13747784; doi:10.3389/fvets.2026.1926523)
Supplement: Supplementary file 4 [file Supplementary_file_4.docx]

# **Supplementary Material S4: Clinical Description of the CNVR Sterilisation Programme**

This supplementary material describes the clinical protocols underpinning the Capture–Neuter–Vaccinate–Release (CNVR) programme evaluated in the main manuscript, including case selection, surgical procedures, anaesthetic and analgesic protocols, vaccination and ear-marking, recovery and release criteria, and the recording of perioperative complications.

## **Selection criteria for surgery**

Prior to capture, field staff conducted a visual health assessment of each animal to determine suitability for surgery. Animals that appeared sick, weak, very underweight, or too young were not collected for sterilisation. Animals presenting with serious illness or injury were instead referred as emergency rescue cases and managed outside the routine CNVR pathway.

## **Surgical procedures**

Ovariohysterectomy (OVH) was performed in females and castration in males. The surgical site was clipped (females: mid-to-caudal abdomen; males: pre-scrotal to scrotal region) and aseptically prepared with a 5-minute chlorhexidine scrub followed by alcohol. During surgery, all four limbs were secured and vital signs (blood pressure, heart rate, and oxygen saturation) were continuously monitored.

## **Anaesthetic and analgesic protocol**

Pre-anaesthetic sedation was achieved with intramuscular xylazine, acepromazine, and tiletamine/zolazepam (Zoletil). Anaesthesia was induced intravenously with propofol (combined with diazepam) and maintained with isoflurane in oxygen. Perioperative analgesia was provided by intramuscular ketoprofen (NSAID). Diluted lidocaine was used for IV administration (female) and a testicular block (male) dogs. Prophylactic antibiotic cover was provided by intravenous cephazolin, and subcutaneous ivermectin was administered as routine antiparasitic treatment.

## **Vaccination and ear-marking**

All animals received a single subcutaneous dose of a combined DHPPi+LR vaccine (Bayovac® DHPPi+LR, Bioveta, a.s., Czech Republic), providing protection against canine distemper, infectious canine hepatitis, infectious canine laryngotracheitis, canine parvovirosis, parainfluenza, leptospirosis (L. icterohaemorrhagiae, L. canicola, L. grippotyphosa), and rabies (inactivated rabies component, min. 2 IU/dose). Per the manufacturer's product information, revaccination is recommended annually for the non-rabies components and biennially (every two years) for the rabies component. Following surgery, all animals were ear-tattooed and/or ear-notched to permanently identify them as sterilised.

## **Recovery and release criteria**

Animals were kept under continuous observation in a dedicated recovery area following surgery. Animals were returned to their point of capture only once fully conscious and ambulatory, with normal respiration, mucous membrane colour, and body temperature.

## **Perioperative complications**

Any complications encountered during surgery (e.g., splenomegaly, pyometra, pregnancy, or cryptorchidism) were recorded on the individual patient record at the time of surgery.
